# Supplementary material for: Integrative Analysis of Metabolome and Transcriptome Reveals the Mechanism of Color Formation in Yellow-Fleshed Kiwifruit
Source: Int J Mol Sci. 2023 Jan 13;24(2):1573. doi: 10.3390/ijms24021573 (PMC9867141; doi:10.3390/ijms24021573)
Supplement: Supplementary file 1 [file ijms-24-01573-s001.zip › Supplementary Figure.pdf]

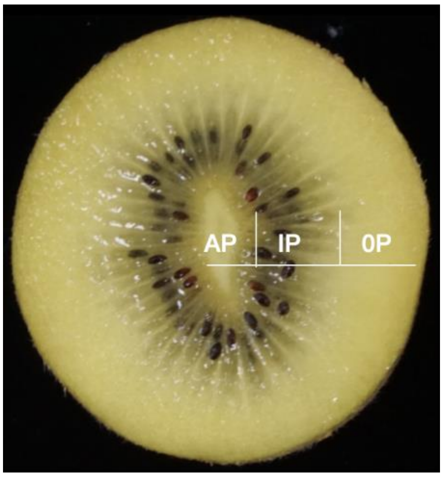

Figure S1. Transverse sections in the kiwifruit. AP, IP, and OP represent axile placentation, inner pericarp, and outer pericarp respectively.

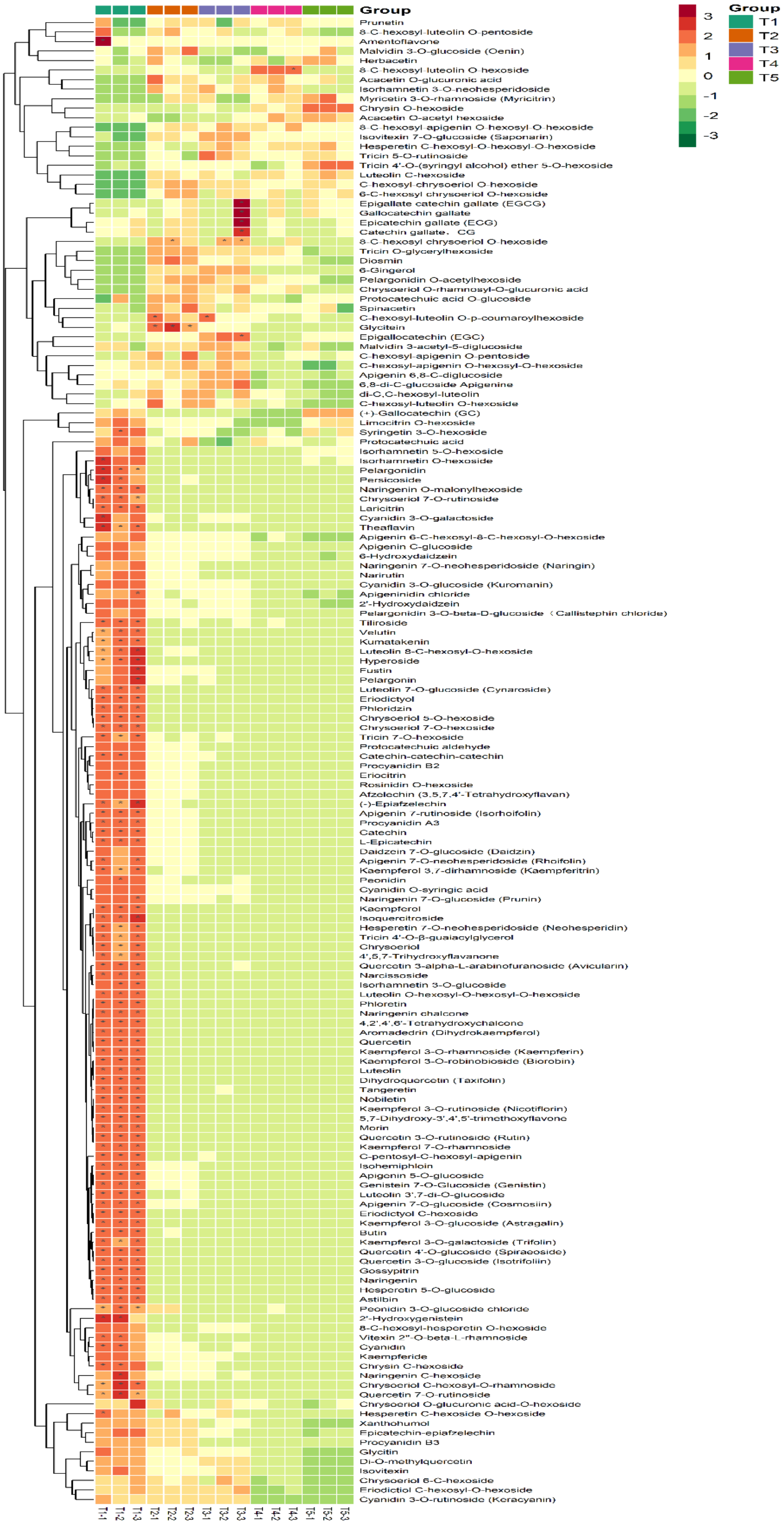

Figure S2. Cluster heat map of relative content of flavonoid.

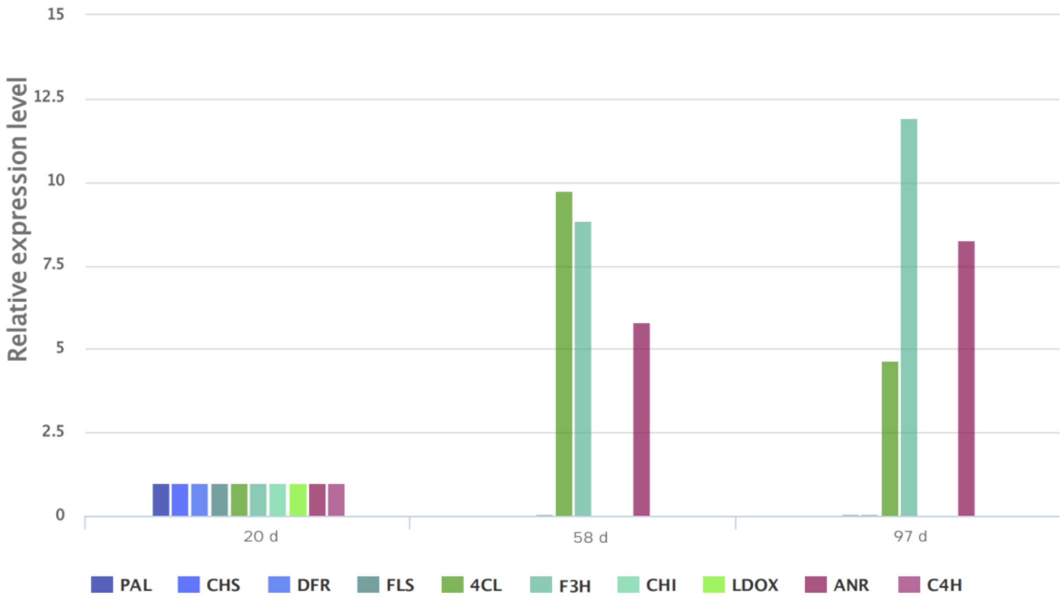

Figure S3. Expressions of the genes *PAL, CHS, DFR, FLS, 4CL, F3H, CHI, LDOX, ANR* and *C4H*, which are associated with anthocyanin biosynthesis. 20 d was set as a reference group in each period, and the gene expression level was recorded as 1. The ordinate value is the multiple of the gene expression amount of each treatment group relative to the gene expression amount of the 20 d, and the scale is logarithmic scale.

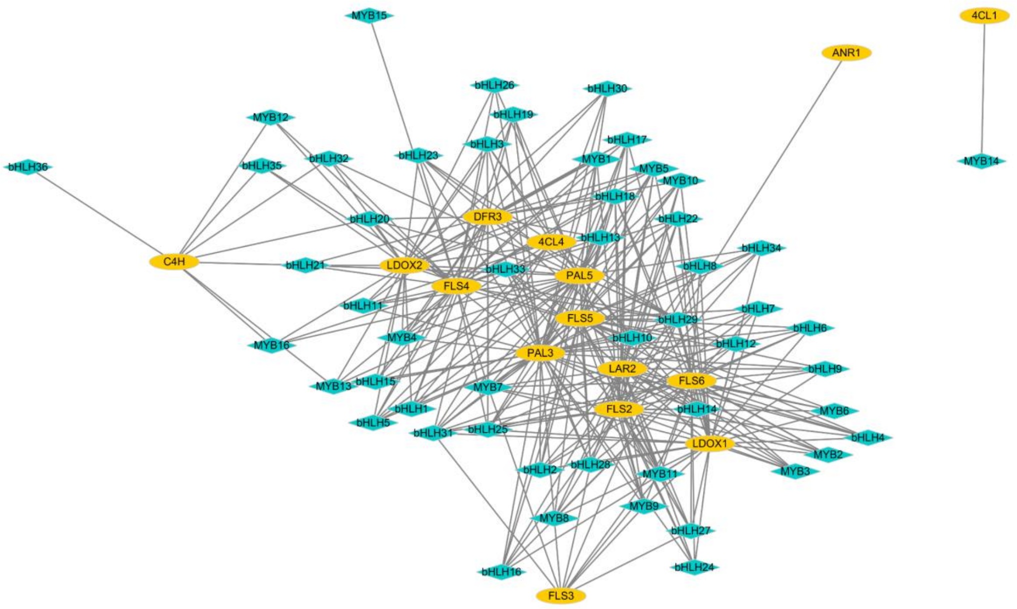

Figure S4. Co-expression network diagram of TFs and structural genes. The circle represents the structural gene, the diamond represents the transcription factor gene, where the node name is customized, the straight line represents the regulatory relationship existing in the gene, and the circle size represents the connectivity (degree) value.

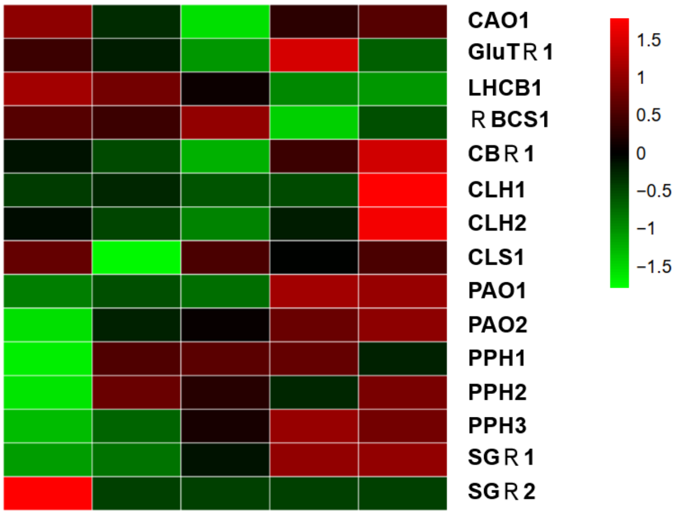

Figure S5. Chlorophyll synthesis and degradation related gene expression level, red represents high expression and green corresponds to low expression.

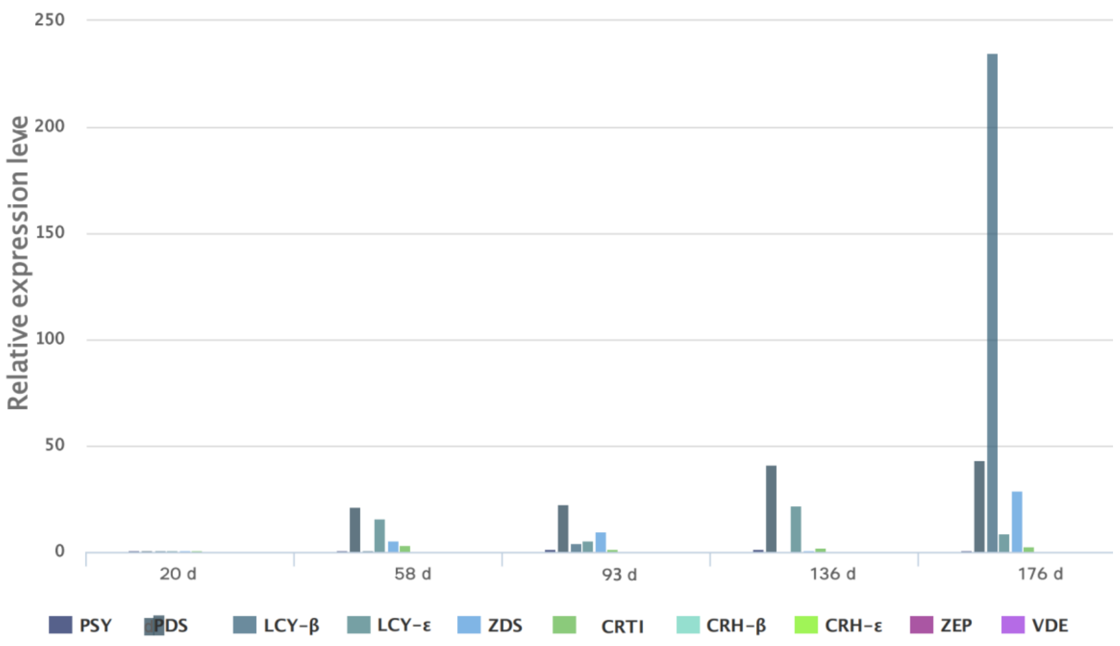

Figure S6. Expressions of the genes *PSY, PDS, LCY-β, LCY-ε, ZDS, CRTISO, CRH-β, CRH-ε, ZEP* and *VDE*, which are associated with carotenoid biosynthesis. 20 d was set as a reference group in each period, and the gene expression level was recorded as 1. The ordinate value is the multiple of the gene expression amount of each treatment group relative to the gene expression amount of the 20 d, and the scale is logarithmic scale.

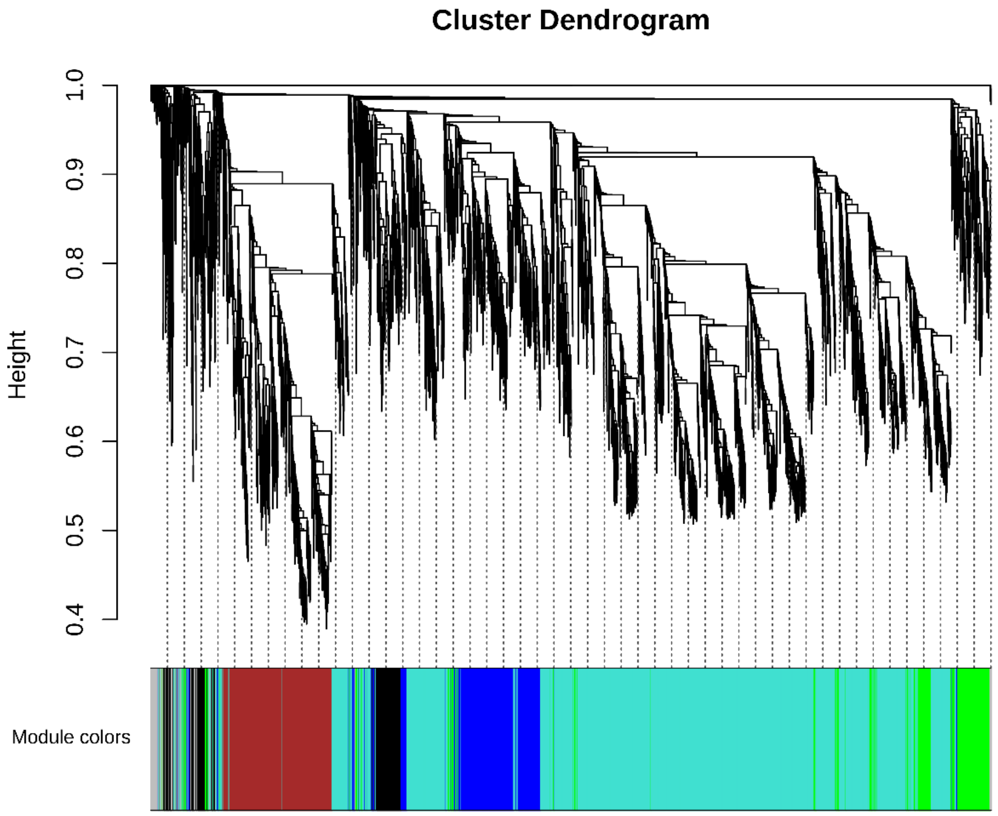

Figure S7. Hierarchical clustering tree (cluster dendrogram) showing 11 modules of coe- xpressed genes by WGCNA. Each leaf of tree corresponds to one gene. The major tree bran- ches constitute 11 modules, labeled with different colors.

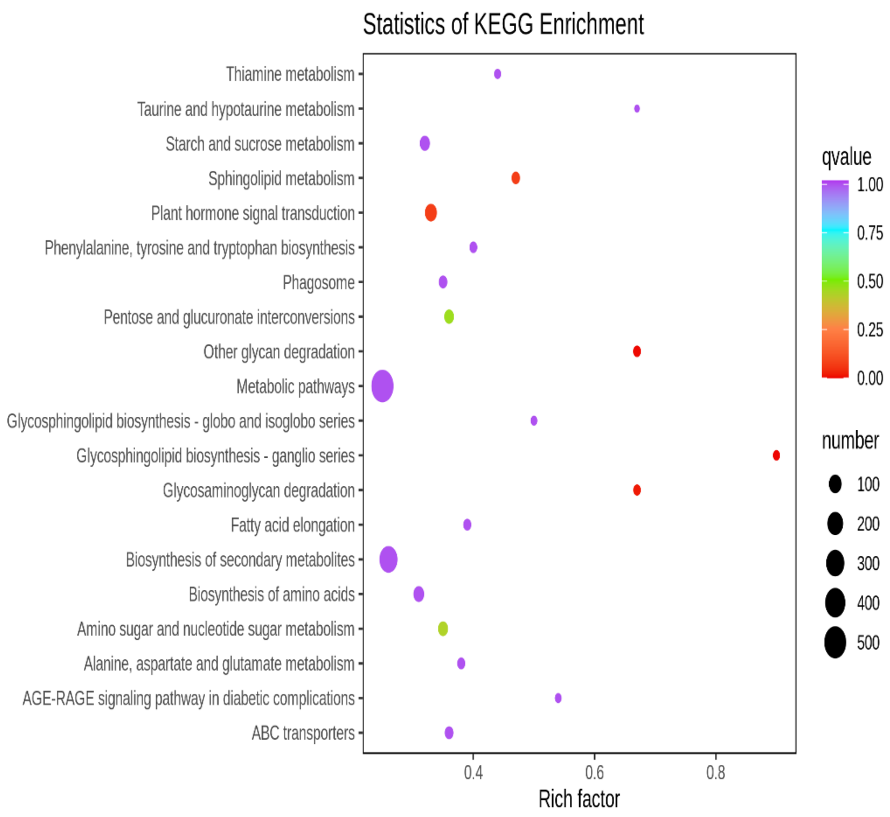

Figure S8. KEGG enrichment analysis of "turquoise" module gene. Modules showed only the top 20 pathways with the most significant enrichment.
